# Control of glioma cell migration and invasiveness by GDF-15

**DOI:** 10.18632/oncotarget.6816

**Published:** 2016-01-04

**Authors:** Paula Codó, Michael Weller, Kerstin Kaulich, Daniel Schraivogel, Manuela Silginer, Guido Reifenberger, Gunter Meister, Patrick Roth

**Affiliations:** ^1^ Laboratory of Molecular Neuro-Oncology, Department of Neurology, University Hospital Zurich and University of Zurich, Zurich, Switzerland; ^2^ Department of Neuropathology, Heinrich Heine University, Düsseldorf, and German Cancer Consortium (DKTK), German Cancer Research Center (DKFZ), Heidelberg, Germany; ^3^ Department of Biochemistry I, University of Regensburg, Regensburg, Germany

**Keywords:** GDF-15, glioblastoma, invasion, TCGA, serpine1

## Abstract

Growth and differentiation factor (GDF)-15 is a member of the transforming growth factor (TGF)-β family of proteins. GDF-15 levels are increased in the blood and cerebrospinal fluid of glioblastoma patients. Using a TCGA database interrogation, we demonstrate that high GDF-15 expression levels are associated with poor survival of glioblastoma patients. To elucidate the role of GDF-15 in glioblastoma in detail, we confirmed that glioma cells express GDF-15 mRNA and protein *in vitro*. To allow for a detailed functional characterization, GDF-15 expression was silenced using RNA interference in LNT-229 and LN-308 glioma cells. Depletion of GDF-15 had no effect on cell viability. In contrast, GDF-15-deficient cells displayed reduced migration and invasion, in the absence of changes in Smad2 or Smad1/5/8 phosphorylation. Conversely, exogenous GDF-15 stimulated migration and invasiveness. Large-scale expression profiling revealed that GDF-15 gene silencing resulted in minor changes in the miRNA profile whereas several genes, including members of the plasminogen activator/inhibitor complex, were deregulated at the mRNA level. One of the newly identified genes induced by GDF-15 gene silencing was the *serpin peptidase inhibitor, clade E nexin group 1* (*serpine1*) which is induced by TGF-β and known to inhibit migration and invasiveness. However, serpine1 down-regulation alone did not mediate GDF-15-induced promotion of migration and invasiveness. Our findings highlight the complex contributions of GDF-15 to the invasive phenotype of glioma cells and suggest anti-GDF-15 approaches as a promising therapeutic strategy.

## INTRODUCTION

Glioblastoma is an aggressive malignancy of the brain characterized by highly infiltrative and rapid growth. Despite multimodal therapy, which includes surgery, radiotherapy and chemotherapy, the median overall survival is limited to approximately 16 months within clinical trials [1]. Therefore, intense efforts for a better understanding of the biology of these tumors are needed to allow for the identification of novel therapeutic targets.

Transforming growth factor (TGF)-β is a major player in the promotion of glioblastoma growth and is the master cytokine within the TGF-β superfamily. We have previously shown that another member of this family, growth and differentiation factor (GDF)-15, also known as macrophage inhibitory cytokine (MIC)-1, contributes to the immune escape of gliomas, a hallmark of these tumors [2]. GDF-15 is synthetized as an inactive pro-protein precursor that is processed by cleavage, folding and dimerization [3] and subsequently secreted. It may then act in a paracrine manner on various cells within the tumor microenvironment [4]. Unprocessed, secreted precursor molecules also bind components of the extracellular matrix, thereby creating latent stromal GDF-15 storages [5].

GDF-15 levels are rather low in normal tissue, with the exception of placenta [6] where GDF-15 exerts immunomodulatory functions. During cancer development and progression, the role of GDF-15 has remained controversial with different findings depending on the tumor entity and models investigated. GDF-15 can promote tumor progression and plays a role in the development of cachexia in late stages of cancer [7, 8]. In the context of glioblastoma, we have characterized GDF-15 as a molecule that contributes to the local immunosuppressive environment surrounding gliomas pointing to a mostly paracrine mode of action [2]. This paracrine function is further supported by reports demonstrating that GDF-15 inhibits immune responses against hepatoma cells [9] and prevents myocardial damage through inhibition of immune cell infiltration following infarction [10]. In contrast, autocrine effects of GDF-15 such as the functional activity of glioma-derived GDF-15 on the tumor cells themselves have remained largely elusive. In the present study, we characterized the autocrine function of GDF-15 using comprehensive approaches including large-scale miRNA and mRNA expression profiling upon gene silencing, thereby aiming at defining the impact of GDF-15 on the malignant phenotype of glioma cells.

## RESULTS

### GDF-15 expression is associated with shorter survival in glioblastoma patients

**Figure 1:**
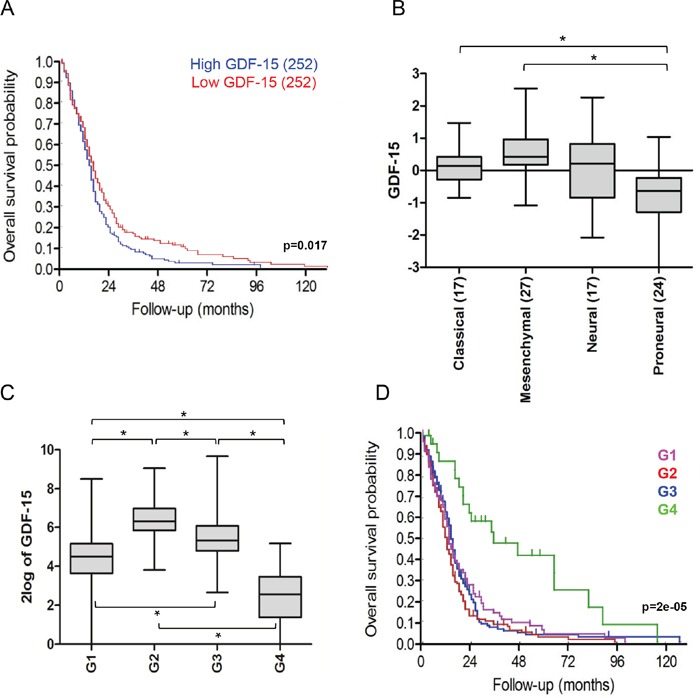
Low GDF-15 expression correlates with better outcome in glioblastoma **A.** TCGA data-based overall survival analysis of patients with tumors with high (blue) versus low (red) GDF-15 mRNA expression (*p* = 0.017). **B.** Relative GDF-15 expression in patient samples stratified according to the Verhaak classification. The number of classified tumors is indicated in brackets (* p < 0.05). **C.** Expression clusters defined by differential GDF-15 levels and related genes according to k-means algorithm (G1, G2, G3, G4) (* p < 0.05). **D.** Overall survival probability for patients from each expression cluster (*p* = 0.000026).

We and others have previously reported that GDF-15 levels are elevated in the blood and cerebrospinal fluid of glioblastoma patients [2, 11]. To assess the overall prognostic role of GDF-15 in glioblastoma, we performed a TCGA database interrogation. When the median was used as cut-off, glioblastoma patients with tumors displaying lower GDF-15 expression had longer overall survival than patients with tumors characterized by GDF-15 expression higher than the median (*p* = 0.017) (Fig. 1A). Moreover, the four glioblastoma subtypes defined by Verhaak and colleagues [12] exhibited different GDF-15 expression patterns, with lowest levels in the proneural subtype (Fig. 1B). When all samples from the selected database were analyzed, we could define four groups (G1, G2, G3, G4), which can be distinguished by their expression of GDF-15 and the associated expression of other genes (Fig. 1C). Within this cluster analysis, the overall survival probability was significantly higher for patients of group G4, which is characterized by the lowest GDF-15 levels (Fig. 1D), corroborating the hypothesis that GDF-15 contributes to the malignant phenotype of glioblastoma.

### GDF-15 expression and gene silencing in glioma cell lines in vitro

**Figure 2:**
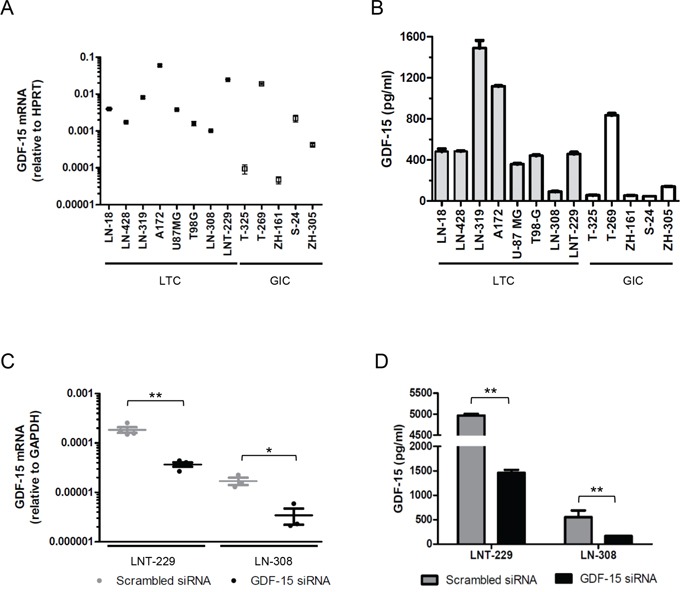
GDF-15 is expressed in glioma cells including GIC and can be silenced by RNA interference **A.** GDF-15 mRNA expression in a panel of LTL and GIC lines was assessed by real-time PCR. **B.** GDF-15 levels in the supernatant of different glioma cell lines were determined by ELISA after cell growth for 48 h. **C.** LNT-229 and LN-308 cells, exposed to GDF-15-specific siRNA oligonucleotides or scrambed siRNA for 72 h, were assessed for GDF-15 expression by real-time PCR. **D.** Cell culture supernatants from the same cells were assessed for GDF-15 protein levels by ELISA (* p < 0.05; ** p < 0.01).

We confirmed GDF-15 mRNA expression in a panel of 8 human long-term glioma cell (LTC) lines and 5 glioma-initiating cell (GIC) lines *in vitro*. There was a trend for higher mRNA and protein levels in LTC than in GIC lines (Fig. 2A,B). The highest GDF-15 protein levels were found in the supernatants of LN-319 and A172 cells among the LTC, and of T-269 cells among the GIC (Fig. 2B). There was a modest correlation between GDF-15 mRNA expression and protein levels in the supernatant (r=0.58, *p* = 0.04). Separate analysis of LTC alone showed no correlation whereas for GIC alone we noticed a strong correlation between mRNA and protein expression (r=0.98, *p* = 0.02). Since hypoxia is a hallmark of glioblastoma, we defined the impact of hypoxic conditions on GDF-15 expression levels *in vitro*. Hypoxia increased GDF-15 mRNA and protein levels in LN-308 cells but not significant change was observed in any other cell line (Suppl. Fig. 1). To allow for a functional characterization of GDF-15 in glioma cells, we silenced GDF-15 expression using RNA interference in LNT-229 and LN-308 cells. Expression analysis 72 h after exposure to specific siRNA oligonucleotides demonstrated a GDF-15 knock-down in the range of 70-80% as assessed by real-time PCR (Fig. 2C) and ELISA (Fig. 2D). Note that GDF-15-silenced LNT-229 cells have still much higher GDF-15 levels than control LN-308 cells. Efforts to silence GDF-15 in other LTC or GIC lines did not result in a sufficient knock-down for further experiments (data not shown).

### Functional consequences of GDF-15 silencing in glioma cells

**Figure 3:**
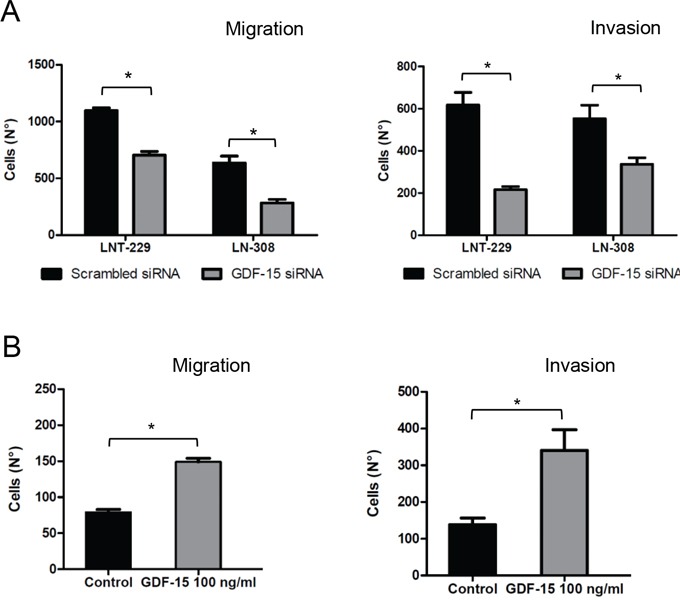
GDF-15 gene silencing reduces glioma cell migration and invasion **A.** GDF-15-depleted cells or control transfectants (24 h) were loaded for transwell migration (left) and Matrigel invasion assays (right). Migration or invasion were assessed at 16 h and 9 fields were counted for every single well. **B.** LNT-229 cells were treated with recombinant GDF-15 as indicated and migration or invasion experiments were performed as in (A) (* p < 0.05).

In order to define autocrine effects of glioma-derived GDF-15 in detail, we examined the effect of transient GDF-15 gene silencing on several key features of LNT-229 and LN-308 glioma cells. Colony formation was not affected by GDF-15 depletion in either cell line (Suppl. Fig. 2A). Next, we analyzed the effects on cell death using annexin V/PI staining 48 h after GDF-15 depletion. No significant differences were detected upon GDF-15 silencing (Suppl. Fig. 2B). However, the migration and invasiveness of GDF-15-depleted LNT-229 and LN-308 cells was significantly reduced (Fig. 3A). Conversely, an increase in cellular migration and invasion was observed when recombinant GDF-15 was added exogenously (Fig. 3B) which stresses the finding that GDF-15 may critically influence these key properties of glioma cells.

### Impact of glioma-derived GDF-15 on the anti-tumor activity of temozolomide and irradiation

The standard of care for glioblastoma patients includes alkylating chemotherapy with temozolomide (TMZ) and irradiation. Based on reports in other tumor entities, we defined the role of GDF-15 in glioma cell sensitivity to these treatments. Cells with transient GDF-15 silencing were exposed to TMZ or irradiated, allowed to grow and analyzed by crystal violet staining for clonogenic survival. GDF-15 gene silencing had no effect on the activity of TMZ (Suppl. Fig. 3A). Similarly, no significant difference was observed between the clonogenic survival of controls and GDF-15-silenced cells after irradiation (Suppl. Fig. 3B). Overall, we concluded that GDF-15 has neither a protective nor a permissive role for TMZ and irradiation treatment *in vitro*.

### Interaction of GDF-15 and TGF-β signaling pathways

**Figure 4:**
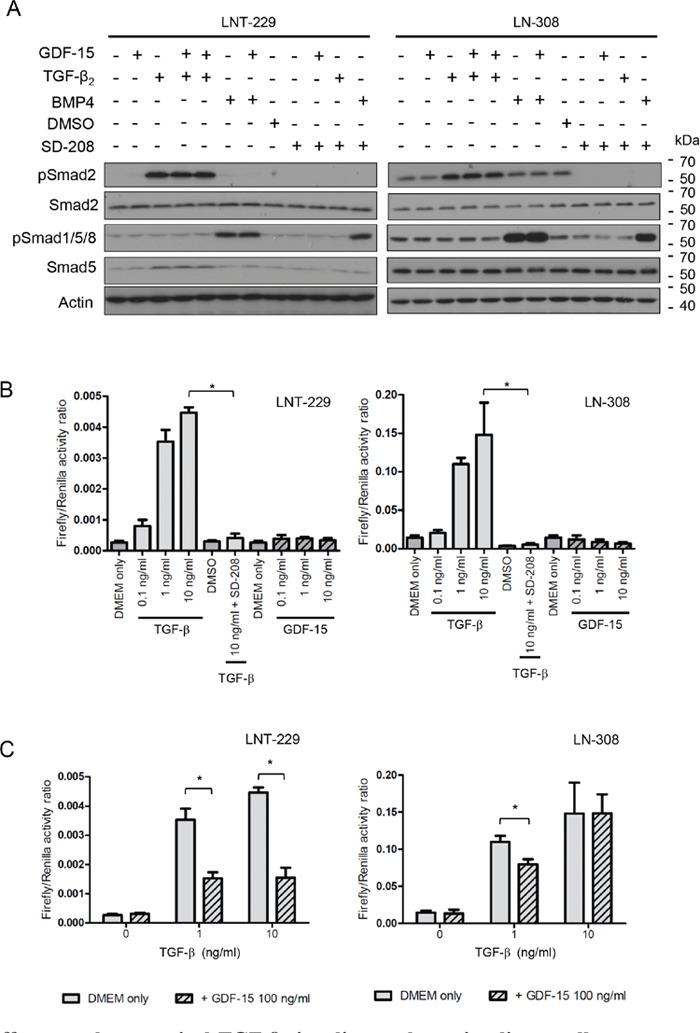
GDF-15 effects on the canonical TGF-β signaling pathway in glioma cells **A.** LNT-229 or LN-308 cells were exposed to GDF-15 (100 ng/ml), BMP-4 (5 ng/ml) or TGF-β_2_ (10 ng/ml) as indicated for 24 h, harvested and assessed for pSmad2, Smad2/3, pSmad1/5/8 and Smad5 levels by immunoblot. **B.** LNT-229 or LN-308 cells transfected with the pGL3-SBE4-Luc reporter plasmid were exposed to TGF-β_2_, GDF-15, SD-208 or combinations thereof as indicated for 24 h, and analyzed for firefly/renilla luciferase activity. **C.** LNT-229 and LN-308 cells transfected with the pGL3-SBE4-Luc reporter plasmid were pre-exposed to GDF-15 for 1.5 h or not as indicated, subsequently treated with TGF-β_2_ for 24 h and analyzed as in (B) (* p < 0.05).

Since GDF-15 is a member of the TGF-β superfamily, we assessed the impact of GDF-15 on the Smad pathway, which reflects the canonical down-stream signaling pathway for TGF-β. In contrast to TGF-β, GDF-15 did not induce Smad2 phosphorylation in any of the cell lines analysed. Furthermore, and different from stimulation with BMP4, there was also no induction of Smad1/5/8 phosphorylation upon GDF-15 exposure (Fig. 4A). We next compared the effects of GDF-15 with those of TGF-β on the activity of the reporter plasmid SBE4-Luc, which contains four copies of the Smad4 binding element. As expected, TGF-β_2_ stimulation resulted in marked reporter gene induction whereas the ALK-5 inhibitor SD-208 abrogated TGF-β_2_–induced reporter activity in LNT-229 and LN-308 cells. No such induction of reporter activity was observed when GDF-15 was added in concentrations ranging from 0.1 to 10 ng/ml. In contrast, the reporter showed even a decreased activity when LN-308 cells were exposed to GDF-15 in a concentration-dependent manner (Fig. 4B). This observation raised the possibility of partial antagonism of TGF-β by GDF-15 at the level of Smad3/Smad4 activation. Indeed, as depicted in Fig. 4C, pre-exposure of glioma cells to GDF-15 resulted in a reduction of the reporter activity in response to TGF-β_2_ in LNT-229 cells. In LN-308 cells, a similar reduction of TGF-β-induced reporter activity was observed, however, only at the lower concentration of TGF-β. Altogether these data point to a modest inhibitory effect of GDF-15 on TGF-β signaling, at the level of Smad3/Smad4 activation in the cell lines investigated.

### GDF-15 silencing affects the transcriptome of glioma cells

In order to gain more insights into the autocrine effects of glioma-derived GDF-15, we assessed the miRNA and mRNA expression profiles upon GDF-15 gene silencing in LNT-229 and LN-308 cells.

**Figure 5:**
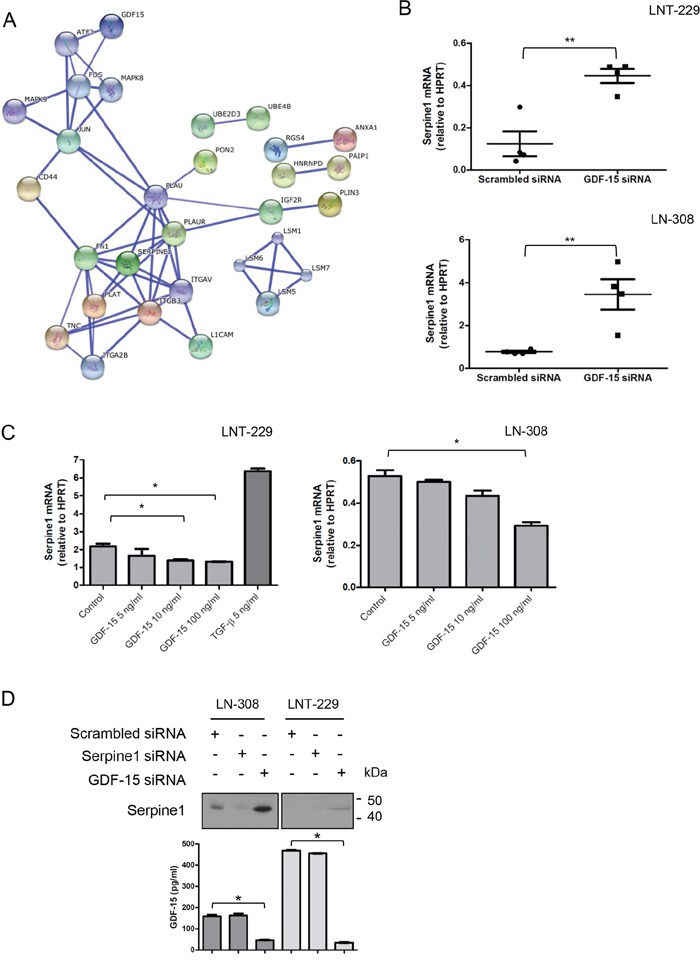
GDF-15 modulates serpine1 expression **A.** STRING analysis of common genes deregulated upon GDF-15 silencing in LNT-229 and LN-308 cell lines (confidence view, score = 0.700, no more than 10 interactions). **B.** Several independent samples of GDF-15-depleted or control transfected LNT-229 or LN-308 cells were analyzed by real-time PCR for serpine1 expression. **C.** The cells were exposed to GDF-15 or TGF-β_2_ as indicated for 24 h and subsequently examined for serpine1 expression by real-time PCR. **D.** Supernatants of cells treated as indicated in (B) for 72 h were assessed for serpine1 levels by immunoblot and GDF-15 concentration by ELISA (* p < 0.05; ** p < 0.01).

As shown in Suppl. Table 1, silencing of GDF-15 resulted in modest changes of the miRNA expression profile of LNT-229 and LN-308 cells. Changes in miRNA expression were represented almost exclusively by an upregulation of miRNA as determined by sequencing. Because of the rather small effects of GDF-15 on the miRNA repertoire, we next aimed at defining the effect of endogenous GDF-15 on the mRNA expression pattern in the same two glioma cell lines, using the Affymetrix GeneChip microarray platform. This analysis identified several transcripts such as members of the plasminogen activator/inhibitor complex, which were regulated in a GDF-15-dependent manner with overall similar changes in LNT-229 and LN-308 cells (Table 1). A bioinformatic analysis revealed that several of the GDF-15-controlled genes are involved in cell motility and metabolism. Further analysis of the mRNA data using the Search Tool for the Retrieval of Interacting Genes/Proteins (STRING) revealed a network of deregulated genes strongly active on the plasminogen activator pathway (Fig. 5A). Because of its central role in this pathway, we confirmed the GDF-15-dependent regulation of *serpin peptidase inhibitor, clade E, member 1* (*serpine1)*, also known as *plasminogen activator inhibitor* (*PAI)-1*, by real-time PCR in both glioma cell lines (Fig. 5B). In line with these findings, addition of exogenous GDF-15 led to decreased serpine1 mRNA expression levels. On the contrary, exposure to recombinant TGF-β induced serpine1 expression (Fig. 5C). Finally, we confirmed the GDF-15-dependent regulation of serpine1 expression at the protein level using immunoblot and ELISA. As shown in Fig. 5D, GDF-15 gene silencing resulted in increased serpine1 secretion into the cell culture supernatant confirming the GDF-15-dependent regulation of serpine1 expression.

**Table 1:** Differential gene expression upon GDF-15 gene silencing in glioma cells

Symbol	Name	LNT-229	LN-308
*SERPINE1*	serpin peptidase inhibitor, clade E (nexin, plasminogen activator inhibitor type 1), member 1	**3.00**	**2.85**
*GDF15*	growth and differentiation factor 15	2.58	5.15
*RGS4*	regulator of G-protein signaling 4	2.54	**1.54**
*ITGB3*	integrin, beta 3 (platelet glycoprotein IIIa, antigen CD61)	**2.16**	**1.42**
*CAPN3*	calpain 3, (p94)	2.02	**1.21**
*MICAL2*	microtubule associated monoxygenase, calponin and LIM domain containing 2	**1.72**	**1.29**
*PLAT*	plasminogen activator, tissue	**1.70**	**1.40**
*TNC*	tenascin C	**1.65**	**1.33**
*PLAUR*	plasminogen activator, urokinase receptor	**1.61**	**1.33**
*L1CAM*	L1 cell adhesion molecule	**1.58**	**1.22**
*PTMA*	prothymosin, alpha	1.66	1.55
*JUN*	jun proto-oncogene	1.48	**1.41**
*SLC2A10*	solute carrier family 2 (facilitated glucose transporter), member 10	**1.45**	**1.47**
*CD59*	CD59 molecule, complement regulatory protein	**1.45**	**1.43**
*ACADVL*	acyl-CoA dehydrogenase, very long chain	**1.43**	**1.37**
*SLC25A46*	solute carrier family 25, member 46	1.41	1.24
*DDR1*	discoidin domain receptor tyrosine kinase 1	**1.40**	**1.21**
*TPST1*	tyrosylprotein sulfotransferase 1	**1.39**	**1.26**
*PON2*	paraoxonase 2	**1.36**	**1.29**
*LEPREL2*	leprecan-like 2	**1.36**	**1.41**
*GNA11*	guanine nucleotide binding protein (G protein), alpha 11 (Gq class)	1.34	1.42
*KYNU*	kynureninase (L-kynurenine hydrolase)	**1.33**	1.06
*HIPK2*	homeodomain interacting protein kinase 2	**1.33**	1.14
*PLIN3*	perilipin 3	**1.31**	**1.15**
*TMPO*	thymopoietin	1.31	1.23
*UBE4B*	ubiquitination factor E4B (UFD2 homolog, yeast)	1.31	1.08
*ANXA1*	annexin A1	**1.30**	**1.11**
*MLLT11*	myeloid/lymphoid or mixed-lineage leukemia (trithorax homolog, Drosophila); translocated to, 11	**1.29**	**1.05**

The most deregulated genes for both cell lines are shown. Bold values imply up-regulation, with fold change values expressed as x=(GDF-15 siRNA/scrambled siRNA). Grey values represents down-regulation and fold change values are expressed as x=(scrambled siRNA/GDF-15 siRNA). Significant genes were included for a p value cut-off of 0.01 for non-paired T test

Since serpine1 is a known TGF-β bona fide target and GDF-15 a member of the TGF-β superfamily, the finding that GDF-15 represses the expression of serpine1 was rather unexpected. In order to examine whether this effect may originate from a direct effect of GDF-15 on the promoter of the *serpine1* gene, we used the p3TP-Lux reporter plasmid, which contains two of the most potent TGF-β responsive elements of the serpine1 promoter [13]. TGF-β_2_, used as a positive control, induced reporter activity and this effect was abrogated by SD-208 in LNT-229 and LN-308 cells. In contrast, exogenous GDF-15 had no significant effect on the baseline reporter gene activity (Suppl. Fig. 4A) and did not interfere with TGF-β-evoked activity (Suppl. Fig. 4B), suggesting that GDF-15 operates either in a different region of the promoter or downstream of transcriptional activation to suppress serpine1 mRNA expression.

### GDF-15-dependent regulation of glioma cell migration is not mediated through serpine1

**Figure 6:**
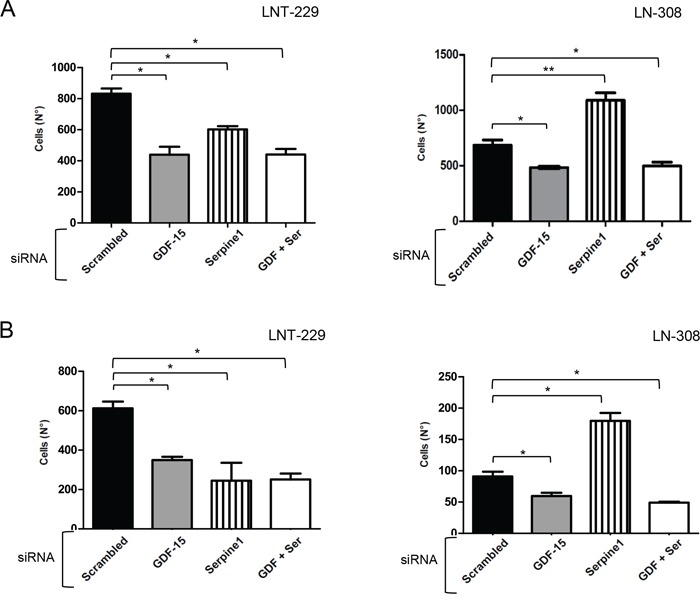
GDF-15 and serpine1 act independently on glioma cell migration and invasion LNT-229 or LN-308 cells were transiently transfected with control siRNA or siRNA molecules targeting GDF-15 or serpine1 as indicated. After 48 h, the cells were used for transwell migration A. or matrigel invasion B. experiments. The assays were stopped after 16 h. Cells present in 9 fields were counted for every single well, 3 wells for each condition (* p < 0.05; ** p < 0.01).

Serpine1 is a secreted protein that inhibits the tissue plasminogen activator (PLAT) and the urokinase-type plasminogen activator receptor (uPAR). uPAR is an important regulator of extracellular matrix (ECM) proteolysis, cell-ECM interactions and cell signaling [14]. We hypothesized that the reduced migration of glioma cells observed upon GDF-15 silencing could be a consequence of increased serpine1 expression. To this end, we transiently silenced GDF-15, serpine1 or both genes simultaneously and analyzed the migration and invasiveness of LNT-229 and LN-308 cells. As shown before, GDF-15 silencing reduced cell migration (Fig. 6A) and invasion (Fig. 6B) in both cell lines, whereas serpine1 gene silencing alone had different effects: while reducing cell migration and invasion in LNT-229, it enhanced both processes in LN-308 cells which may reflect the higher endogenous serpine1 levels in these cells. The combined silencing of GDF-15 and serpine1 showed no difference in migration and invasion when comparing with GDF-15 silencing alone in both cell lines, indicating that the effect observed in cell motility upon silencing GDF-15 is not mediated through serpine1.

## DISCUSSION

Increased GDF-15 levels have been found in the blood of glioblastoma patients [2] and in the CSF where it correlates with poor patient outcome [11]. However, there are also reports suggesting that GDF-15 may act as a tumor suppressor [15, 16]. Here, we aimed at clarifying the biological role of glioma-derived GDF-15 in more detail. Analysis of the gene expression dataset deposited in the TCGA database demonstrated that high GDF-15 levels are associated with reduced overall survival of glioblastoma patients (Fig. 1A). Further analyses showed that GDF-15 is also differentially expressed in the context of the transcriptional glioblastoma subgroups described by Verhaak et al. [12]: proneural, neural, classical and mesenchymal. The reasons for the differential expression of GDF-15 within the 4 subgroups remain speculative. Highest GDF-15 levels were found in the mesenchymal subgroup which may respond best to various immunotherapeutic strategies that are currently under development [17, 18]. Thus, targeting GDF-15 and thereby abrogating its immunosuppressive properties may be a potentially beneficial therapeutic strategy in this molecular subgroup. When all dataset samples were grouped in 4 clusters, regardless of their subtype classification but according to their GDF-15 expression levels, we noticed that the group of patients whose tumors had the lowest GDF-15 expression levels had prolonged survival (Fig. 1D). Although requiring confirmation in another dataset with control for other prognostic factors, these findings corroborate previous reports indicating that GDF-15 contributes to the malignant phenotype of glioblastoma [2, 11, 19]. Hypoxia resulted in increased GDF-15 levels in LN-308 cells which supports previous findings [19]. However, no effect of hypoxic conditions was observed in various other glioma cell lines suggesting that hypoxia might not be a major driver of GDF-15 expression.

GDF-15 gene silencing using RNA interference did not reduce glioma cell viability (Suppl. Fig. 2). Of note, we used only a transient silencing of GDF-15 and it remains unclear whether a stable GDF-15 gene silencing would have resulted in different effects on clonogenic survival. Most importantly, two key characteristics of glioma cells, migration and invasion, were impaired upon GDF-15 depletion. In line with these findings, the addition of recombinant GDF-15 enhanced cell migration and invasion (Figs. 3A and 3B) indicating that autocrine GDF-15 signaling plays an important functional role in glioma cells. The contribution of GDF-15 to the invasiveness of cancer cells has also been described in the context of gastric cancer cells [20]. Furthermore, it was reported that GDF-15 protects prostate cancer [21] and colon cancer [22, 23] cells from the deleterious effects of chemotherapeutic agents. In contrast to these findings, we did not observe any change in the susceptibility of glioma cells to the alkylating agent, TMZ, or to irradiation (Suppl. Fig. 3).

Since a receptor for GDF-15 has not yet been characterized, there is no consensus on the signaling pathway for this molecule under physiological and pathological conditions [4]. Because GDF-15 belongs to the TGF-β superfamily, we aimed at disclosing whether any of the canonical signaling pathways described for this family, which are represented by the activation of Smad2/3 or Smad1/5/8 [24, 25], are affected by GDF-15. Exposure of LNT-229 or LN-308 cells to recombinant GDF-15 did not trigger Smad2 or Smad1/5/8 phosphorylation, a finding that is in line with previous reports in gastric cancer cells [20]. Furthermore, pSmad3/Smad4 complex activation was not observed upon GDF-15 stimulation as assessed using the SBE4 gene reporter [13]. On the contrary, we noticed a reduction of the basal reporter activity in LN-308 cells when GDF-15 was applied (Fig. 4B). Competition experiments demonstrated a reduced reporter activity in response to TGF-β in the presence of GDF-15 in LNT-229, and the same at low concentrations of TGF-β in LN-308 cells. Since LNT-229 cells are characterized by low endogenous TGF-β_2_ activity, we concluded that GDF-15-mediated interference with canonical TGF-β signaling may only occur when this pathway is not strongly activated.

To increase the understanding of autocrine GDF-15 effects in glioma cells, we analyzed miRNA and mRNA expression profiles by sequencing and array technology, respectively. Although GDF-15 had different effects on the miRNA expression in the 2 cell lines investigated (Suppl. Table 1 ), there were overall only minor changes indicating that the observed functional consequences of GDF-15 gene silencing are probably not mediated at the level of miRNA function. More pronounced changes were noticed at the mRNA level upon GDF-15 depletion. When analyzing the dataset using the STRING algorithm for gene interaction visualization, we detected that several genes were clustered around the plasminogen activation pathway: plasminogen activator urokinase type (PLAU), PLAU receptor (PLAUR), PLAT, tenascin (TNC), serpine1, as well as genes directly involved in cell motility such as members of the mitogen activated protein kinase (MAPK) pathway. Unexpectedly, the expression of serpine1, the most deregulated gene, is inhibited by GDF-15 in contrast to the effect of TGF-β, a well-known inducer of serpine1 expression [26]. GDF-15-dependent regulation of serpine1 expression was confirmed in independent experiments at the transcriptional and protein levels. We elaborated on this in more detail and used the p3TP-Lux reporter plasmid [13] which contains the most potent TGF-β responsive element of the human serpine1 promoter and has been widely used as a TGF-β reporter system. While exposure to TGF-β resulted in increased reporter activity, no response was recorded upon GDF-15 administration. Furthermore, GDF-15 did not abrogate TGF-β-mediated induction of reporter gene activity suggesting that the inhibitory effect of GDF-15 on serpine1 mRNA expression might be due to repression of the serpine1 gene in other regions of the promoter not being part of the reporter plasmid or mediated through other posttranscriptional mechanisms that cause mRNA instability.

Upon secretion to the extracellular medium, serpine1 interacts with soluble PLAT and the extracellular domain of uPAR, inhibiting their serin-protease activity and ultimately resulting, among other effects, in reduced activity of matrix metalloproteinases (MMP) [14, 27]. MMP can degrade ECM components and release growth factors retained within the ECM such as TGF-β [28, 29]. Furthermore, uPAR is an important regulator of ECM proteolysis, cell-ECM interactions and cell signaling involved in actin cytoskeleton remodeling [14, 30, 31]. Accordingly, we hypothesized that GDF-15-mediated down-regulation of serpine1 levels may enhance the activity of this cascade resulting in increased tumor cell motility. However, when GDF-15 and serpine1 were silenced, either alone or in combination, we did not recognize a clear link between GDF-15, serpine1 and the observed phenotype (Fig. 6). Of note, silencing of serpine1 alone had different effects on the invasion and migration of LNT-229 and LN-308 glioma cells. This might be explained by the different constitutive expression levels and functional relevance of serpine1 in the two cell lines.

In conclusion, we delineate GDF-15 as a molecule that promotes migration and invasion of glioma cells suggesting a relevant contribution to the malignant phenotype of these tumors. Furthermore, GDF-15 has been described as an important immunosuppressive cytokine in the context of gliomas and other pathological conditions [2, 9, 10, 32]]. In light of the various immunotherapeutic approaches which are currently being investigated in glioblastoma patients, the autocrine and paracrine functions of GDF-15 may make it an attractive target for novel therapeutic strategies.

## MATERIALS AND METHODS

### Cells and reagents

The human LTC lines A172, U87MG and T98G were purchased from the American Type Culture Collection. LN-18, LNT-229, LN-308, LN-319 and LN-428 cells were kindly provided by Dr. N. de Tribolet (Lausanne, Switzerland). LTC were maintained in Dulbecco's modified eagle medium (DMEM, Invitrogen, Life Technologies, Carlsbad, CA), containing 10% fetal calf serum (FCS) (VWR Lonza, Leighton Buzzard, UK) and supplemented with 2 mM glutamine (Invitrogen, Life Technologies), in a 5% CO_2_ incubator at 37° C. The GIC lines S-24, T-269, T-325, ZH-161 and ZH-305 were generated and cultured as described [33–35]. GIC were maintained as sphere cultures in Neurobasal A medium (Invitrogen, Life Technologies) supplemented with EGF (10 ng/ml), FGF (10 ng/ml) (Peprotech, Rocky Hill, NJ), heparin (31.5 U/ml) (Sigma Aldrich, St. Louis, MO), 1% Glutamax (Invitrogen, Life Technologies) and 2% B27 (Invitrogen, Life Technologies). Adherent cells were detached with TrypLE Express (Invitrogen, Life Technologies). For experiments under hypoxic conditions, the cells were cultured in a hypoxia chamber adjusted to 5% CO_2_ and 1% O_2_ at 37°C.

Recombinant human TGF-β_2_ (rh-TGF-β_2_) and BMP-4 (rh-BMP4) were purchased from R&D Systems (Minneapolis, MN), recombinant human GDF-15 (rh-GDF-15) from Peprotech. The following antibodies were used for immunoblot experiments: rabbit anti-human phospho-Smad2 (Ser465/467) (clone 138D4, Cell Signaling Technology, Danvers, MA), rabbit anti-human Smad2 (clone 86F7, Cell Signaling Technology), rabbit anti-human phospho-Smad1(ser463/465)/Smad-5(ser463/465)/Smad8(Ser426/428, Cell Signaling Technology), rabbit anti-human Smad5 antibody (Cell Signaling Technology), goat anti-human actin (Santa Cruz Biotechnology, Dallas, TX), goat anti-human serpine1 (Acris Antibodies, Inc, San Diego, CA). The TGF-β receptor I kinase inhibitor SD-208 was kindly provided by Scios Inc (Fremont, CA). Temozolomide (TMZ) was provided by Schering-Plough (Kenilworth, NJ).

### Gene expression and Kaplan-Meier analysis of survival probability using The Cancer Genome Atlas (TCGA) dataset

Overall survival analysis within the TCGA database (http://cancergenome.nih.gov), for glioblastoma 540 - MAS5.0 - u133a dataset (*n* = 540) was performed using the Kaplan-Meier analysis module of the R2: microarray analysis and visualization platform (http://r2.amc.nl). The scan cut-off mode based on median GDF-15 expression was selected without specifying a track subset. Analysis of GDF-15 expression by glioblastoma subtype [12] was done using 85 samples classified into these groups within the subtype track mode and z-score transformation. Generation of GDF-15 expression clusters across the whole 540—MAS5.0 dataset was performed with k-means algorithm and 2Log transformation of gene expression. Kaplan-Meier overall survival curves were plotted for the generated clusters.

### GDF-15 enzyme-linked immunosorbent assay (ELISA)

1.5 × 10^6^ glioma cells were seeded in a 5 cm dish or a T25 flask in complete medium and allowed to attach or form spheres for 24 h. Medium was subsequently changed to FCS- or B27-free medium. Supernatants were harvested and stored at −80°C until further assessment by ELISA (R&D Systems). Total protein concentration for normalization was determined using the Pierce™ BCA Protein Assay Kit (Thermo Scientific, Lafayette, CO).

### RNA interference-mediated gene silencing

Glioma cells were seeded at proper density for each cell line on a six-well plate or 6 cm tissue culture dishes (TPP, Trasadingen, Switzerland), allowed to attach overnight and transfected with ON-TARGET plus human GDF-15 siRNA SMARTpool or ON-TARGET plus non-targeting pool (Dharmacon/Thermo Scientific, Lafayette, CO). The transfection reagent used was Metafectene® Pro (Biontex, Switzerland). Nine hours after transfection, the medium was changed to FCS-free medium. Cells and supernatants were harvested at 24, 48 or 72 h.

### Real-time polymerase chain reaction

Total RNA was prepared using the NucleoSpin® kit (Macherey Nagel GmbH, Düren, Germany). Complementary DNA was prepared using the iScript® kit (BioRad, Hercules, CA). For real-time PCR, gene expression was measured in an Applied Biosystems 7300 Real-Time PCR System using the ABI Prism 7000 Sequence Detection System (Applied Biosystems, Carlsbad, CA) with SYBR Green ROX Mix (Thermo Scientific) and primers (Microsynth AG, Balgach, Switzerland) at a final concentration of 0.4 μM. GAPDH and GDF-15 primers have been described [2, 36]. Other primer sequences were: serpine1 fwd: 5′-GCTTACAGGAGCTTTTGTGT-3′, rev: 5′-ACTCTGAGATGAAAGGGTGTTT-3′, HPRT fwd: 5′-TGAGGATTTGGAAAGGGTGT-3′, rev: 5′-GAGCACACAGAGGGCTACAA-3′. The conditions were 40 cycles at 95°C/15 s and 60°C/1 min. Standard curves were generated for each gene and the amplification was 90–100% efficient. Relative quantification of gene expression was determined by comparison of threshold values. Results were normalized to HPRT and calculated with the ΔCT method for relative quantification [37].

### Clonogenic survival assay

Clonogenic assays were performed by seeding 500 cells per well in 96-well plates. After overnight attachment, the cells were exposed to different agents as indicated for 24 h, in FCS-free medium in case of TMZ, followed by observation for 7–14 days in complete medium. Cell density was assessed by crystal violet staining.

### Flow cytometry

For cell death analysis, cells were detached with Accutase™ (PAA, Pasching, Austria) and 5 × 10^5^ cells were used per staining. Cells re-suspended in Annexin buffer [10 mM HEPES, 140 nM NaCl, 2.5 mM CaCl_2_, pH= 7.4] were stained with Annexin-V-FITC (BD Biosciences) and propidium iodide (PI) (Sigma-Aldrich) for 15 min at room temperature in the dark and analyzed by flow cytometry.

### Migration and invasion

Cells with transient GDF-15 gene silencing, control transfectants or cells pre-treated with 100 ng/ml rh-GDF-15 for 24 h were detached, washed and re-suspended in serum-free medium alone or supplemented with rh-GDF-15. A cell suspension containing 50000 cells was added to the upper well of transwell migration inserts (pore size: 8 μm, BD Biosciences) or to BD BioCoat™ Matrigel™ invasion chambers (pore size: 8 μm, BD Biosciences). In the lower well, 700 μl of complete medium were used as chemo-attractant. The cells were kept for 16 h at 37°C and 5% CO_2_, followed by fixation in cold methanol for 10 min and staining with Mayer's alum haematoxylin for 1 h. Inserts were mounted in glass slides and 9 fields per sample were counted, 3 replicates of each treatment.

### Immunoblot analysis

The cells were lysed in radioimmunoprecipitation assay (RIPA) buffer [10 mM Tris pH 8.0, 150 mM NaCl, 1% NP-40, 0.5% deoxycholate, 0.1% SDS] supplemented with 1% complete protease inhibitor mix (Roche Diagnostics, Basel, Switzerland) and phosphatase inhibitor cocktails 1 and 2 (Sigma-Aldrich). Serum-free conditioned media were concentrated using Centriplus Centrifugal Filter Device YM-3 (3000 Da cut-off; Millipore, Billerica HQ, MA). Protein concentration was measured with the Bradford protein assay reagent (Bio-Rad) using bovine serum albumin as a standard. Samples were mixed with Laemmli buffer containing 10% β-mercaptoethanol (BioRad). Twenty μg of protein were loaded per lane, in a 12% poly acrylamide gel. Protein transfer was performed using nitrocellulose 45 μm pore size (BioRad). Equal protein loading was ascertained by actin detection. Visualization of protein bands was accomplished using horseradish peroxidase–coupled secondary antibodies (Sigma-Aldrich) diluted 1:5000 with enhanced chemoluminescence (ECL, Thermo Scientific).

### Luciferase-gene reporter assay

The pGL3-SBE4-Luc reporter plasmid was kindly provided by Dr. Bert Vogelstein (Baltimore, MD). This reporter has 4 copies of the Smad4 binding site of Vestigial (Vg) Drosophila gene palindrome sequences on the promoter of firefly luciferase, cloned in the pBV-Luc vector [38]. The pGL2-3TP-Luc construct contains a synthetic promoter composed of a TGF-β-responsive fragment of the promoter of the human plasminogen activator inhibitor-1 (PAI-1 or serpine1) gene inserted downstream of three phorbol ester-responsive elements [13], kindly provided by J. Massague (New York, NY). Dual luciferase firefly/renilla assays were performed with co-transfection of 150 ng of the respective reporter construct and 20 ng of pRL-CMV VECTOR (Promega, Madison, WI). Luciferase activity was normalized to constitutive renilla luciferase activity (pRL-CMV).

### Microarray-based gene expression profiling

GDF-15-dependent gene regulation was examined in LNT-229 and LN-308 glioma cells. To this end, the cells were transfected with GDF-15-specific siRNA oligonucleotides or scrambled control. Total RNA was extracted with the RNeasy kit (Qiagen, Hilden, Germany) 72 h after transfection. Gene expression profiling was performed using hybridization of Affymetrix Gene Chip HG-U133 Plus 2.0 arrays (Affymetrix, Santa Clara, CA). High quality of the extracted RNA (RNA integrity number > 9.0) was assured by using the Agilent 2100 Bioanalyzer (Agilent Technologies, Böblingen, Germany). The Affymetrix 3′IVT express kit was used for sample preparation and labeling starting with 100 ng total RNA. After fragmentation, labeled cRNA was hybridized to the microarrays for 16 h at 42°C, stained with strepatavidin/phycoerythrin conjugate, and scanned. Data analyses on Affymetrix CEL files were conducted with the GeneSpring GX software (Vers. 12.1; Agilent Technologies). Probes within each probe set were summarized by robust multi-array analysis (RMA). Signal intensities were quantile-normalized across all samples to reduce inter-array variability. Input data pre-processing was concluded by baseline transformation of each probe set to the median of all samples. After grouping of samples siGDF-15 versus siScrambled, a given transcript had to be expressed above background (i.e. fluorescence signal of a given probe set was detected within the 20^th^ and 100^th^ percentiles of the signal distribution of a given array) in the three biological replicates to be further analyzed. Significant differential gene expression was determined using unpaired t-tests.

### miRNA expression profiling

miRNA expression profiling was done with LNT-229 and LN-308 cells with a silenced GDF-15 gene or control cells as described above. Total RNA was extracted from the cells using TRIzol reagent (Life Technologies). Biological duplicates were processed from control or GDF-15 silenced LNT-229 and LN-308 cells.

Size selection for 21 nt small RNA (sRNA) fraction was done from 10 μg total RNA separated on a 12% urea PAGE. After gel run, a piece corresponding to 19 to 24 nt RNA was excised and RNA was eluted from gel overnight by passive diffusion into elution buffer [300 mM NaCl, 2 mM EDTA] at 25°C. RNA was precipitated from eluate with 75% ethanol overnight and dissolved in water. Small RNA cloning from the sRNA fraction was essentially done as described before [39] using 75% of the isolated sRNA fraction. Cluster generation, sequencing and FastQ file generation were carried out at the sequencing core facility Center of Excellence for Fluorescent Bioanalytics KFB at the University of Regensburg. Cluster generation was done with TruSeq SR Cluster Kit v3 on a cBOT (Illumina, San Diego, CA). Sequencing was performed on a HiScan SQ (Illumina) using TruSeq SBS v3 in 50 bp single-end runs with 10 samples per lane. FastQ files were generated by CASAVA 1.8.

Deep sequencing data were processed with in house software and annotated against *Homo sapiens* miRBase Version 19 without any allowed mismatches. Only miRNA with more than 20 reads per million reads (rpmr) as mean value over all samples were included into analysis.

### Search tool for the retrieval of interacting Genes/proteins (STRING) analysis

The interactome of mRNA which are regulated in a GDF-15-dependent manner in LNT-229 and LN-308 cells was delineated using the STRING 9.1 platform (http://string-db.org; [40]. Interactions with a confidence score of 0.7 or higher were integrated using all available active prediction methods.

### Statistics

Data are expressed as mean and standard error of the mean. The experiments shown were repeated at least three times with similar results. Data are shown as a representative result. Analysis of significance for overall survival probability curves was done using the Cochran-Armitage comparison for Pearson's chi-square fit. For analysis of GDF-15 expression levels in the TCGA datasets, ANOVA (Kruskal-Wallis test) with Dunn's multiple comparison test was applied. For all other data, unpaired, two-tailed Student's t-test were performed (GraphPad Prism 5, La Jolla, CA) (*p < 0.05, **p < 0.01).

## SUPPLEMENTARY FIGURES AND TABLE





## References

[1] Weller M, van den Bent M, Hopkins K, Tonn JC, Stupp R, Falini A, Cohen-Jonathan-Moyal E, Frappaz D, Henriksson R, Balana C, Chinot O, Ram Z, Reifenberger G (2014). EANO guideline for the diagnosis and treatment of anaplastic gliomas and glioblastoma. Lancet Oncol.

[2] Roth P, Junker M, Tritschler I, Mittelbronn M, Dombrowski Y, Breit SN, Tabatabai G, Wick W, Weller M, Wischhusen J (2010). GDF-15 contributes to proliferation and immune escape of malignant gliomas. Clin Cancer Res.

[3] Bauskin AR, Zhang HP, Fairlie WD, He XY, Russell PK, Moore AG, Brown DA, Stanley KK, Breit SN (2000). The propeptide of macrophage inhibitory cytokine (MIC-1), a TGF-beta superfamily member, acts as a quality control determinant for correctly folded MIC-1. EMBO J.

[4] Mimeault M, Batra SK (2010). Divergent molecular mechanisms underlying the pleiotropic functions of macrophage inhibitory cytokine-1 in cancer. J Cell Physiol.

[5] Bauskin AR, Jiang L, Luo XW, Wu L, Brown DA, Breit SN (2010). The TGF-beta superfamily cytokine MIC-1/GDF15: secretory mechanisms facilitate creation of latent stromal stores. J Interferon Cytokine Res.

[6] Segerer SE, Rieger L, Kapp M, Dombrowski Y, Muller N, Dietl J, Kammerer U (2012). MIC-1 (a multifunctional modulator of dendritic cell phenotype and function) is produced by decidual stromal cells and trophoblasts. Hum Reprod.

[7] Breit SN, Carrero JJ, Tsai VW, Yagoutifam N, Luo W, Kuffner T, Bauskin AR, Wu L, Jiang L, Barany P, Heimburger O, Murikami MA, Apple FS (2012). Macrophage inhibitory cytokine-1 (MIC-1/GDF15) and mortality in end-stage renal disease. Nephrol Dial Transplant.

[8] Malyszko J, Koc-Zorawska E, Malyszko JS, Glowinska I, Mysliwiec M, Macdougall IC (2013). GDF15 is related to anemia and hepcidin in kidney allograft recipients. Nephron Clin Pract.

[9] Zhou Z, Li W, Song Y, Wang L, Zhang K, Yang J, Zhang W, Su H, Zhang Y (2013). Growth differentiation factor-15 suppresses maturation and function of dendritic cells and inhibits tumor-specific immune response. PLoS One.

[10] Kempf T, Zarbock A, Widera C, Butz S, Stadtmann A, Rossaint J, Bolomini-Vittori M, Korf-Klingebiel M, Napp LC, Hansen B, Kanwischer A, Bavendiek U, Beutel G (2011). GDF-15 is an inhibitor of leukocyte integrin activation required for survival after myocardial infarction in mice. Nat Med.

[11] Shnaper S, Desbaillets I, Brown DA, Murat A, Migliavacca E, Schluep M, Ostermann S, Hamou MF, Stupp R, Breit SN, de Tribolet N, Hegi ME (2009). Elevated levels of MIC-1/GDF15 in the cerebrospinal fluid of patients are associated with glioblastoma and worse outcome. Int J Cancer.

[12] Verhaak RG, Hoadley KA, Purdom E, Wang V, Qi Y, Wilkerson MD, Miller CR, Ding L, Golub T, Mesirov JP, Alexe G, Lawrence M, O'Kelly M (2010). Integrated genomic analysis identifies clinically relevant subtypes of glioblastoma characterized by abnormalities in PDGFRA, IDH1, EGFR, and NF1. Cancer Cell.

[13] Wrana JL, Attisano L, Carcamo J, Zentella A, Doody J, Laiho M, Wang XF, Massague J (1992). TGF beta signals through a heteromeric protein kinase receptor complex. Cell.

[14] Smith HW, Marshall CJ (2010). Regulation of cell signalling by uPAR. Nat Rev Mol Cell Biol.

[15] Yoshioka H, Kamitani H, Watanabe T, Eling TE (2008). Nonsteroidal anti-inflammatory drug-activated gene (NAG-1/GDF15) expression is increased by the histone deacetylase inhibitor trichostatin A. J Biol Chem.

[16] Kadowaki M, Yoshioka H, Kamitani H, Watanabe T, Wade PA, Eling TE (2012). DNA methylation-mediated silencing of nonsteroidal anti-inflammatory drug-activated gene (NAG-1/GDF15) in glioma cell lines. Int J Cancer.

[17] Doucette T, Rao G, Rao A, Shen L, Aldape K, Wei J, Dziurzynski K, Gilbert M, Heimberger AB (2013). Immune heterogeneity of glioblastoma subtypes: extrapolation from the cancer genome atlas. Cancer Immunol Res.

[18] Weiss T, Weller M, Roth P (2015). Immunotherapy for glioblastoma: concepts and challenges. Curr Opin Neurol.

[19] Albertoni M, Shaw PH, Nozaki M, Godard S, Tenan M, Hamou MF, Fairlie DW, Breit SN, Paralkar VM, De Tribolet N, Van Meir EG, Hegi ME (2002). Anoxia induces macrophage inhibitory cytokine-1 (MIC-1) in glioblastoma cells independently of p53 and HIF-1. Oncogene.

[20] Lee DH, Yang Y, Lee SJ, Kim KY, Koo TH, Shin SM, Song KS, Lee YH, Kim YJ, Lee JJ, Choi I, Lee JH (2003). Macrophage inhibitory cytokine-1 induces the invasiveness of gastric cancer cells by up-regulating the urokinase-type plasminogen activator system. Cancer Res.

[21] Mimeault M, Johansson SL, Batra SK (2013). Marked improvement of cytotoxic effects induced by docetaxel on highly metastatic and androgen-independent prostate cancer cells by downregulating macrophage inhibitory cytokine-1. Br J Cancer.

[22] Whiteside MA, Chen DT, Desmond RA, Abdulkadir SA, Johanning GL (2004). A novel time-course cDNA microarray analysis method identifies genes associated with the development of cisplatin resistance. Oncogene.

[23] Proutski I, Stevenson L, Allen WL, McCulla A, Boyer J, McLean EG, Longley DB, Johnston PG (2009). Prostate-derived factor--a novel inhibitor of drug-induced cell death in colon cancer cells. Mol Cancer Ther.

[24] Schmierer B, Hill CS (2007). TGFbeta-SMAD signal transduction: molecular specificity and functional flexibility. Nat Rev Mol Cell Biol.

[25] Pickup M, Novitskiy S, Moses HL (2013). The roles of TGFbeta in the tumour microenvironment. Nat Rev Cancer.

[26] Keeton MR, Curriden SA, van Zonneveld AJ, Loskutoff DJ (1991). Identification of regulatory sequences in the type 1 plasminogen activator inhibitor gene responsive to transforming growth factor beta. J Biol Chem.

[27] Wilkins-Port CE, Ye Q, Mazurkiewicz JE, Higgins PJ (2009). TGF-beta1 + EGF-initiated invasive potential in transformed human keratinocytes is coupled to a plasmin/MMP-10/MMP-1-dependent collagen remodeling axis: role for PAI-1. Cancer Res.

[28] Dallas SL, Rosser JL, Mundy GR, Bonewald LF (2002). Proteolysis of latent transforming growth factor-beta (TGF-beta )-binding protein-1 by osteoclasts. A cellular mechanism for release of TGF-beta from bone matrix. J Biol Chem.

[29] Gomez-Duran A, Mulero-Navarro S, Chang X, Fernandez-Salguero PM (2006). LTBP-1 blockade in dioxin receptor-null mouse embryo fibroblasts decreases TGF-beta activity: Role of extracellular proteases plasmin and elastase. J Cell Biochem.

[30] Czekay RP, Loskutoff DJ (2004). Unexpected role of plasminogen activator inhibitor 1 in cell adhesion and detachment. Exp Biol Med (Maywood).

[31] Czekay RP, Aertgeerts K, Curriden SA, Loskutoff DJ (2003). Plasminogen activator inhibitor-1 detaches cells from extracellular matrices by inactivating integrins. J Cell Biol.

[32] Wu A, Wei J, Kong LY, Wang Y, Priebe W, Qiao W, Sawaya R, Heimberger AB (2010). Glioma cancer stem cells induce immunosuppressive macrophages/microglia. Neuro Oncol.

[33] Rieger J, Lemke D, Maurer G, Weiler M, Frank B, Tabatabai G, Weller M, Wick W (2008). Enzastaurin-induced apoptosis in glioma cells is caspase-dependent and inhibited by BCL-XL. J Neurochem.

[34] Weiler M, Blaes J, Pusch S, Sahm F, Czabanka M, Luger S, Bunse L, Solecki G, Eichwald V, Jugold M, Hodecker S, Osswald M, Meisner C (2014). mTOR target NDRG1 confers MGMT-dependent resistance to alkylating chemotherapy. Proc Natl Acad Sci U S A.

[35] Codo P, Weller M, Meister G, Szabo E, Steinle A, Wolter M, Reifenberger G, Roth P (2014). MicroRNA-mediated down-regulation of NKG2D ligands contributes to glioma immune escape. Oncotarget.

[36] Carraro G, Albertin G, Forneris M, Nussdorfer GG (2005). Similar sequence-free amplification of human glyceraldehyde-3-phosphate dehydrogenase for real time RT-PCR applications. Mol Cell Probes.

[37] Roth P, Silginer M, Goodman SL, Hasenbach K, Thies S, Maurer G, Schraml P, Tabatabai G, Moch H, Tritschler I, Weller M (2013). Integrin control of the transforming growth factor-beta pathway in glioblastoma. Brain.

[38] Zawel L, Dai JL, Buckhaults P, Zhou S, Kinzler KW, Vogelstein B, Kern SE (1998). Human Smad3 and Smad4 are sequence-specific transcription activators. Mol Cell.

[39] Dueck A, Ziegler C, Eichner A, Berezikov E, Meister G (2012). microRNAs associated with the different human Argonaute proteins. Nucleic Acids Res.

[40] Szklarczyk D, Franceschini A, Kuhn M, Simonovic M, Roth A, Minguez P, Doerks T, Stark M, Muller J, Bork P, Jensen LJ, von Mering C (2011). The STRING database in 2011: functional interaction networks of proteins, globally integrated and scored. Nucleic Acids Res.

