# Supplementary material for: Control of glioma cell migration and invasiveness by GDF-15
Source: Oncotarget. 2016 Jan 4;7(7):7732–46. doi: 10.18632/oncotarget.6816 (PMC4884950; doi:10.18632/oncotarget.6816)
Supplement: Supplementary file 1 [file oncotarget-07-7732-s001.pdf]

SUPPLEMENTARY FIGURES AND TABLE

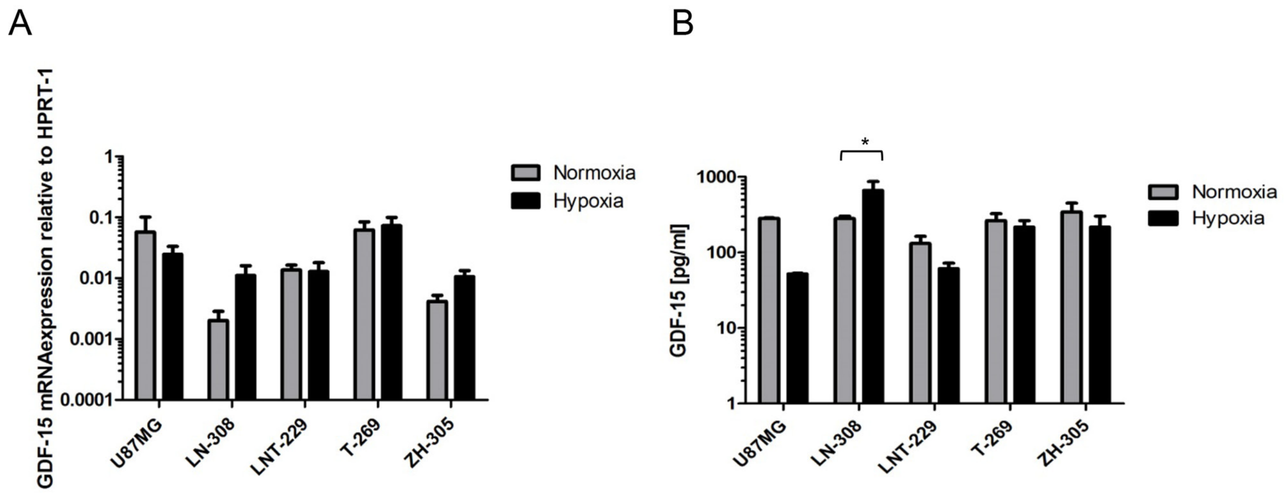

Supplementary Figure S1: Effects of hypoxia on GDF-15 expression. A. GDF-15 mRNA expression levels in a panel of LTC and GIC lines exposed to normoxia or hypoxia (1% O_2) for 24 h were assessed by real-time PCR. **B.** GDF-15 protein levels in the supernatant of different glioma cell lines were assessed by ELISA after cell growth under normoxic or hypoxic conditions for 48 h (*$p < 0.05$).

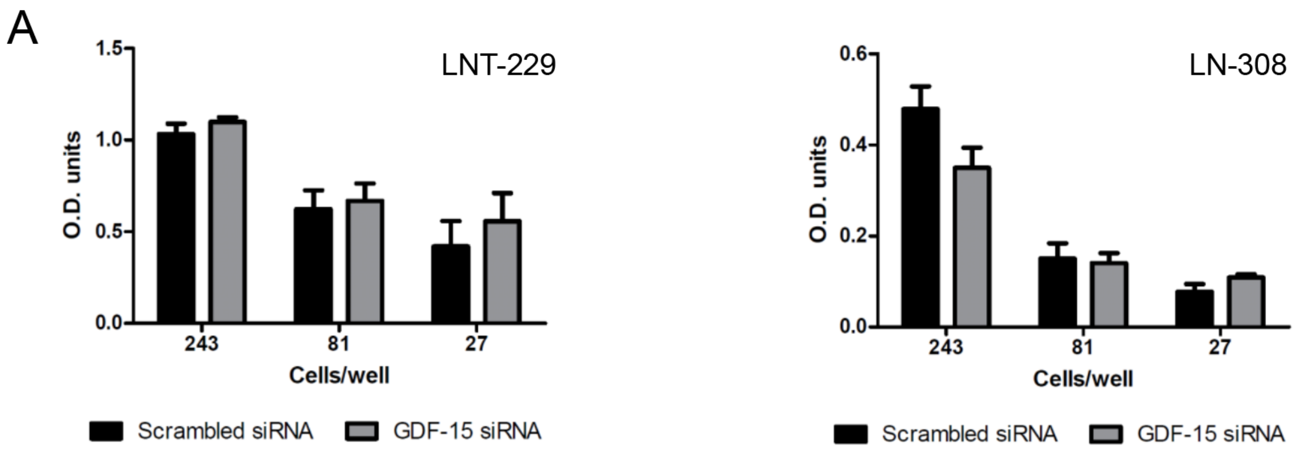

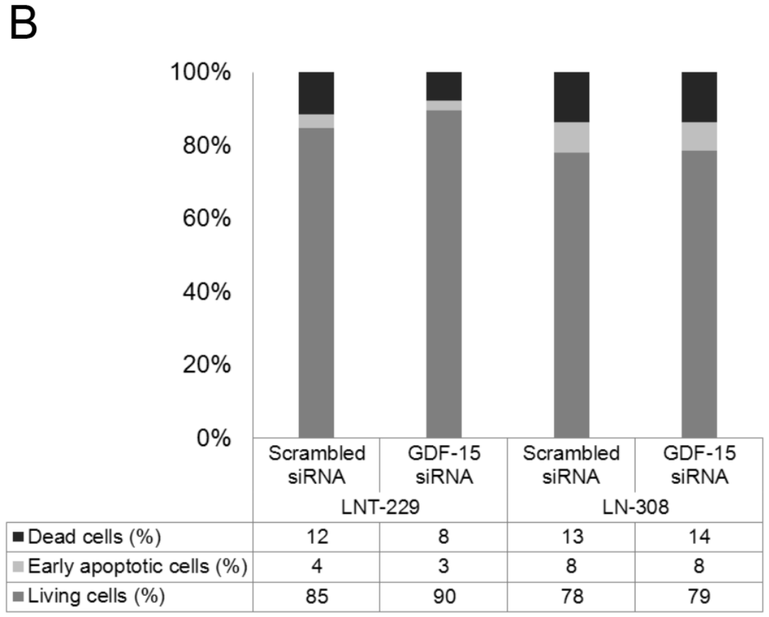

	Scrambled siRNA	GDF-15 siRNA	Scrambled siRNA	GDF-15 siRNA
	LNT-229		LN-308	
■ Dead cells (%)	12	8	13	14
■ Early apoptotic cells (%)	4	3	8	8
■ Living cells (%)	85	90	78	79

Supplementary Figure S2: GDF-15 gene silencing has no effect on cell viability. A. GDF-15-depleted or control cells were seeded in 96-well plates and allowed to grow 2–3 weeks in complete medium before colony formation was assessed by crystal violet staining. **B.** The viability of glioma cells with a silenced GDF-15 gene or control cells was assessed by flow cytometry using annexin V/PI staining 48 h after siRNA exposure.

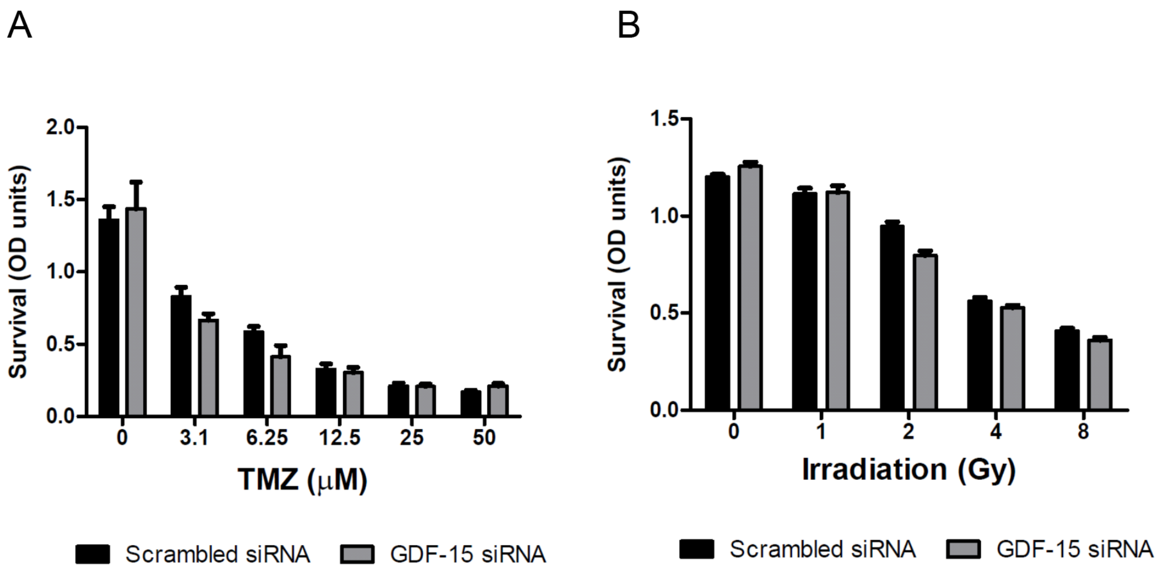

Supplementary Figure S3: GDF-15 gene silencing has no effect on glioma cell sensitivity to TMZ or irradiation *in vitro*. GDF-15-depleted or control LNT-229 cells were exposed to various concentrations of TMZ **A.** or irradiated at different doses **B.** as indicated. Subsequently, the cells were allowed to grow for 2–3 weeks and analyzed by crystal violet staining (*$p < 0.05$; **$p < 0.01$).

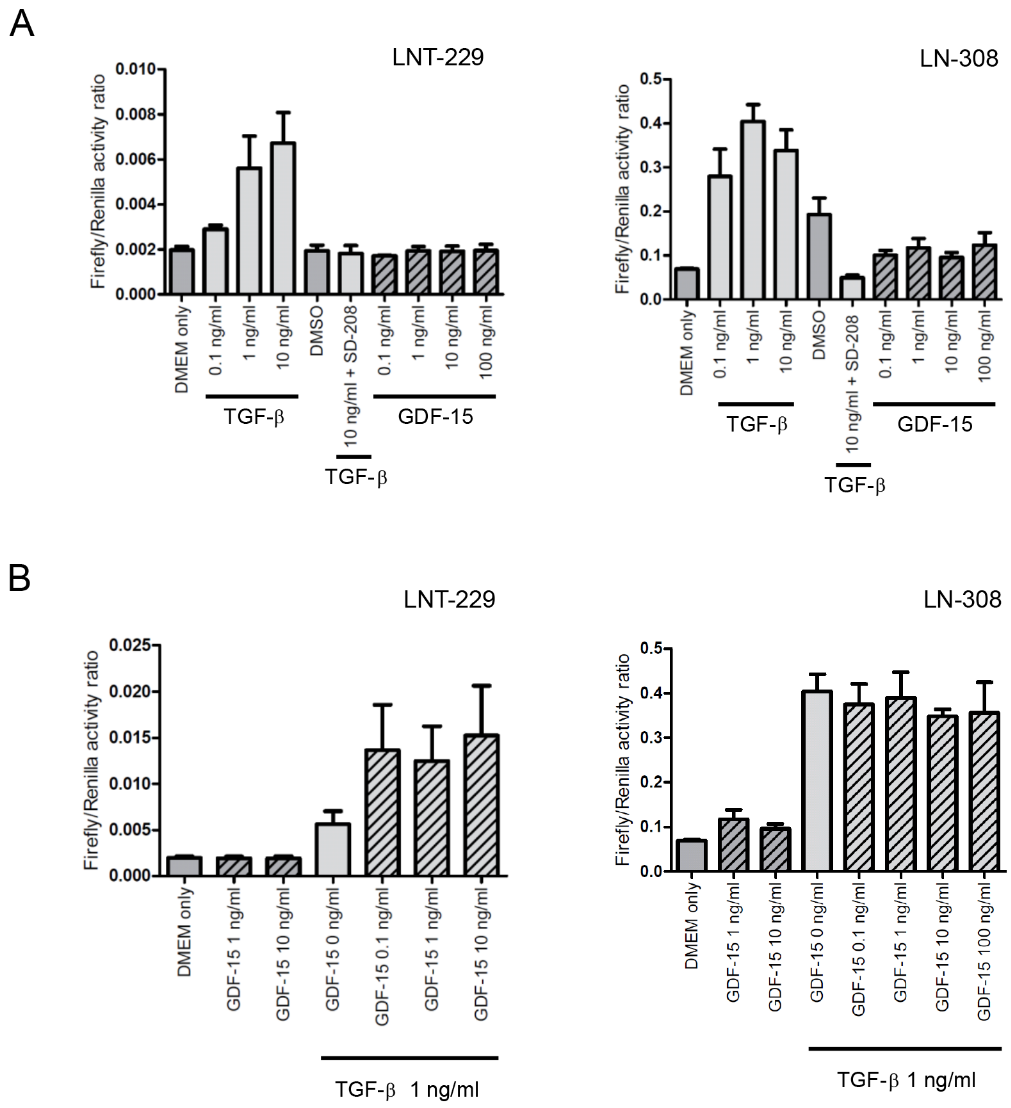

Supplementary Figure S4: Effects of GDF-15 and TGF-β on the p3TP-Lux reporter. A. LNT-229 or LN-308 cells were transiently transfected with the p3TP-Lux reporter plasmid and exposed to TGF-β_2, GDF-15, SD-208 or combinations thereof as indicated for 24 h and subsequently analyzed for firefly/renilla luciferase activity. **B.** LNT-229 or LN-308 cells were transiently transfected with the p3TP-Lux reporter plasmid, pre-exposed to GDF-15 as indicated for 1.5 h, treated with TGF-β_2 for 24 h and analyzed as in (A) (*$p < 0.05$; **$p < 0.01$).

Supplementary Table S1: Changes in the miRNA expression profile in GDF-15-depleted glioma cells. GDF-15-depleted LNT-229 and LN-308 were analyzed for their miRNA repertoire by deep sequencing and compared to control transfectants. The table shows logarithmic fold changes of GDF-15 knock-down (kd) versus control (ctrl) samples and read numbers as reads per million reads (rpmr) over both biological replicates from either LNT-229 or LN-308 cells

See Supplementary Table 1
